# Cytokine Effects on the Entry of Filovirus Envelope Pseudotyped Virus-Like Particles into Primary Human Macrophages

**DOI:** 10.3390/v11100889

**Published:** 2019-09-23

**Authors:** Tzanko S. Stantchev, Autumn Zack-Taylor, Nicholas Mattson, Klaus Strebel, Christopher C. Broder, Kathleen A. Clouse

**Affiliations:** 1 Division of Biotechnology Review and Research 1 (DBRR1), Office of Biotechnology Products (OBP), Center for Drug Evaluation and Research (CDER), US Food and Drug Administration (FDA), Silver Spring, MD 20993, USA; autumn.zack-taylor@fda.hhs.gov (A.Z.-T.); nicholas.mattson@fda.hhs.gov (N.M.); 2 Laboratory of Molecular Microbiology, National Institute of Allergy and Infectious Diseases (NIAID), National Institutes of Health (NIH), Bethesda, MD 20814, USA; kstrebel@niaid.nih.gov; 3 Department of Microbiology and Immunology, Uniformed Services University of the Health Sciences (USUHS), Bethesda, MD 20814, USA; christopher.broder@usuhs.edu

**Keywords:** Ebola virus (EBOV), filoviruses, cytokines, interleukin-10 (IL-10)

## Abstract

Macrophages are one of the first and also a major site of filovirus replication and, in addition, are a source of multiple cytokines, presumed to play a critical role in the pathogenesis of the viral infection. Some of these cytokines are known to induce macrophage phenotypic changes in vitro, but how macrophage polarization may affect the cell susceptibility to filovirus entry remains largely unstudied. We generated different macrophage subsets using cytokine pre-treatment and subsequently tested their ability to fuse with beta-lactamase containing virus-like particles (VLP), pseudotyped with the surface glycoprotein of Ebola virus (EBOV) or the glycoproteins of other clinically relevant filovirus species. We found that pre-incubation of primary human monocyte-derived macrophages (MDM) with interleukin-10 (IL-10) significantly enhanced filovirus entry into cells obtained from multiple healthy donors, and the IL-10 effect was preserved in the presence of pro-inflammatory cytokines found to be elevated during EBOV disease. In contrast, fusion of IL-10-treated macrophages with influenza hemagglutinin/neuraminidase pseudotyped VLPs was unchanged or slightly reduced. Importantly, our in vitro data showing enhanced virus entry are consistent with the correlation established between elevated serum IL-10 and increased mortality in filovirus infected patients and also reveal a novel mechanism that may account for the IL-10-mediated increase in filovirus pathogenicity.

## 1. Introduction

Filoviruses, particularly members of the Ebola and Marburg genera, cause severe disease with one of the highest mortality rates observed among human pathogens. Increased levels of a variety of cytokines/chemokines, presumed to be an integral part of virus pathogenesis, have been established in patients infected with strains of the *Zaire* [1,2,3,4,5,6,7,8,9], *Sudan* [10] and/or *Bundibugyo* [11] *ebolavirus* species.

It was demonstrated in vitro that not only *ebolavirus* infection [12,13,14,15], but also *ebolavirus* surface glycoprotein(s) (GP), presented as either soluble molecules or on the surface of replication incompetent virus-like particles or inactivated virions [14,16,17,18,19,20,21,22,23,24,25,26], and as *ebolavirus* GP derived peptides [12,18,26,27,28], are capable of inducing cytokine expression in challenged cells. Several cell surface molecules have been implicated in the EBOV GP—induced cytokine secretion (including IL-10), Tim-1 [29], TLR-4 [30,31] and/or LSECtin (CLEC4G)/DAP12 [18]. The role of Tim-1 and TLR-4 was demonstrated using a transgenic mouse model, while the effect(s) of LSECtin/DAP12 were established in human monocyte-derived dendritic cells.

Despite species to species, strain to strain and study to study variability, the EBOV and Sudan virus (SUDV) strains were generally found to induce a variety of pro-inflammatory cytokines (IL-1β, IL-6, IL-8, MCP-1, MIP-1α, MIP-1β, etc.,) both in vivo and in vitro. Some [1,2,3,9], but not all [6,8], studies reported significantly increased TNF-α levels in samples from EBOV infected patients. TNF-α was not increased in the serum samples of patients infected with SUDV, although several other pro-inflammatory cytokines (IL-6, IL-8, IP-10, MIP-1β [10], IL-1α, IL-6, IP-10, MCP-1, MIP-1α [32]) were elevated in non-survivors. From pro-inflammatory cytokines/chemokines, only MCP-1 serum levels were increased in Bundibugyo virus (BDBV)- infected individuals [11] and peripheral blood mononuclear cells (PBMC) infected with BDBV produced 2- to 10-fold lower levels of TNF-α, MCP-1, IL-1β and MIP1-α compared to cells infected with EBOV strain Mayinga [13].

The role of pro-inflammatory cytokines in filovirus pathogenesis appears to be complex and may be time-dependent. Although higher levels of pro-inflammatory cytokines are generally associated with increased viral load and/or poor prognosis [1,7,8,9,32,33,34,35,36], there are also data suggesting that an early robust pro-inflammatory cytokine response may result in asymptomatic infection [4,5] or improved survival [2].

Intriguingly, the anti-inflammatory cytokine IL-10 appears to be consistently elevated in patients infected with EBOV [1,2,3,8,9,33], SUDV [10] and BDBV [11], and has been associated with poor disease outcome.

IL-4 and IL-13, which may also be referred to as anti-inflammatory cytokines [37], have been less extensively studied in EBOV disease (EVD). Waquier; et al., [35] reported either similar or significantly lower IL-4 and/or IL-13 levels, compared to healthy controls, in samples obtained from surviving and non-surviving patients during the outbreaks in Gabon and the Republic of Congo, which occurred between 1996 and 2005. Increased IL-13 (days 7 and 8 of clinical illness), but undetectable IL-4 levels, were established in a healthcare care worker infected with the Makona strain during the 2014–2016 EBOV outbreak [6]. Transient elevations of IL-4 were detected in several Makona-infected patients during the course of disease but, in general, these increases were not considered statistically significant [8].

Macrophages are one of the major filovirus targets and serve both as sites of virus replication and sources of cytokine production (reviewed in [6,25,38,39]). However, there is a paucity of information as to how the altered cytokine levels in EVD may affect the susceptibility of uninfected macrophages to virus entry at the sites of filovirus replication. Currently, it is well established in vitro that macrophages may present as heterogeneous phenotypes, driven by certain cytokines. In brief, TNF-α is known to induce the classical activated M1 phenotype, while IL4 and/or IL-13 induce the alternatively activated M2a macrophages and IL-10 treatment is associated with cell polarization to the M2c phenotype (reviewed in [40,41,42,43,44]). In this study, we pre-incubated macrophages with these cytokines and tested their ability to support the entry of β-lactamase (BLaM)-containing virus-like particles (VLP), pseudotyped with different filovirus surface GPs. To mimic more closely the in vivo conditions, we also performed the same experiments using macrophages pre-incubated with a cocktail(s) of cytokines known to be elevated in EVD. Using macrophages from multiple healthy donors, we established that pre-incubation with IL-10, alone or in combination with pro-inflammatory cytokines, resulted in increased filovirus GP pseudotyped VLP entry, while having an opposite or no effect on the fusion of VLP pseudotyped with influenza hemagglutinin (HA)/neuraminidase (NA). Subsequently, by performing microarray and Flow cytometry analysis, we attempted to identify the IL-10 inducible factors potentially affecting the filovirus VLP entry into primary macrophages.

## 2. Materials and Methods

### 2.1. Reagents

Recombinant cytokines were purchased from Peprotech (Rocky Hill, NJ, USA) and/or R&D Systems (Minneapolis, MN, USA). The cytokines were re-suspended in 0.5% clinical grade human serum albumin (HSA) to a concentration of 20 mg/mL and used immediately or aliquoted and stored at –80 °C. Dulbecco’s Modified Eagle’s Medium (DMEM), Penicillin/Streptomycin antibiotic solution, sodium pyruvate (100 mM), Dulbecco’s Phosphate Buffered Saline (DPBS) with or without Ca^2+^ and Mg^2+^, and L-glutamine (200 mM) were obtained from Lonza (Walkersville, MD, USA). Phenol red-free DMEM and the CCF2/AM Beta lactamase Loading Kit (GeneBLAzer Reporter Assay) were purchased from Invitrogen/Thermo Fisher Scientific (Carlsbad, CA, USA). HEPES buffer solution (1 M) was purchased from Quality Biological (Gaithersburg, MD, USA). The nuclear fluorescent dye DRAQ5^TM^ was obtained from BioStatus Shepshed, UK, or Thermo Fisher Scientific. The FuGENE 6 transfection reagent was purchased from Promega Corp. (Madison, WI, USA). Probenecid was obtained from Sigma-Aldrich (St. Louis, MO, USA), Enzo Life Sciences (Farmingdale, NY, USA) or Thermo Fisher Scientific (Waltham, MA, USA). HSA (25% sterile solution) was purchased from CSL Behring (Kankakee, IL, USA). The pooled human serum was prepared in house from multiple serum aliquots, obtained at the NIH Department of Transfusion Medicine from healthy donors, seronegative for HIV-1, HIV-2, hepatitis B and hepatitis C.

**Antibodies:** The APC conjugated anti-CD206 (Clone 19.2), anti-IL21R (Clone 17A12) and anti-CD29/integrin β1 (Clone MAR4) monoclonal antibodies (mAb), and the FITC labeled anti-CD61/integrin β3 mAb were obtained from BD Pharmingen. The anti- CD163 PE labeled mAb (clone GHI/61), the anti-Niemann-Pick cholesterol transporter 1 (NPC1) (Clone 17B5) and anti-cathepsin L (Clone 33/1) mAb (unlabeled or FITC conjugated) were obtained from Invitrogen/Thermo Fisher Scientific. The anti-human CD51/integrin alpha V/CD51 (Clone P2W7) Alexa Fluor^®^ 488-conjugated mAb was purchased from R&D Systems

**Plasmids:** The plasmids VP40, VP40-BlaM and VP40-GFP were provided by Dr. Yoshihiro Kawaoka, Dr. Lijun Rong and Dr. Paul Bieniasz, respectively. The plasmids encoding the full length (VRC6001) and the Δ-mucin form (VRC6002) of the Kikwit strain EBOV GP were provided by Dr. Gary Nabel. The plasmids encoding the full-length surface glycoproteins of EBOV strain Mayinga, Sudan virus (SUDV) strain Boniface, SUDV strain Gulu_,_ Bundibugyo virus (BDBV), Tai Forest virus (TAFV), Reston virus (RESTV) and Marburg virus (MARV) strain Angola were provided by Dr. Andrea Marzi. The plasmid encoding the Makona strain EBOV GP (GeneBank: KX013101.1) was designed in our laboratory using the VectorBuilder (Cyagen) software and subsequently synthesized de novo by VectorBuilder. The RBR-Fc protein and/or the RBR-Fc expression plasmid were provided by Dr. Judith White. The RBR-Fc protein was expressed in 293T cells, purified by Protein A affinity chromatography and concentrated using Millipore 10 kDa cut off pore size centrifugal filter units. The Vpr-BlaM encoding plasmid was provided by Dr. Mike Miller and the psPAX2 packaging plasmid was obtained from Addgene (Addgene plasmid 12260, a gift from Dr. Didier Trono). The influenza hemagglutinin (HA) [A/Vietnam 2004 (H5N1)] and neuraminidase (NA) [A/California 2009 (H1N1)] encoding pVRC/R plasmids were provided by Dr. Carol Weiss.

### 2.2. Cells and Cell Culture Conditions

The 293T cells were obtained from Dr. G. Quinnan (USUHS, Bethesda, MD) and maintained in DMEM supplemented with 10% fetal calf serum (FCS), 2 mM L-glutamine, and antibiotics (DM-10) at 37 °C in a humidified, 5% CO2 atmosphere. Human peripheral blood mononuclear cells (PBMC) were isolated by Ficoll-Paque^TM^ gradient centrifugation following leukapheresis of healthy seronegative donors. Monocytes and lymphocytes were further separated using countercurrent centrifugal cell elutriation as previously described [45]. Macrophages were prepared from elutriated monocytes by differentiation in 100 mm square Petri dishes (Bibbi Sterilin Ltd., Stone Staffs, UK) in DMEM supplemented with 10% human serum pooled from multiple donors, 2 mM L-glutamine and antibiotics (MØ medium) [46,47]. After 7 to 14 days of differentiation in the absence of exogenous growth factors, the monocyte-derived macrophages (MDM) were detached (by incubation in DBPS at 4 °C) and plated immediately in 96 and/or 48 well plates in the presence of TNF-α, IL-4, IL-10 or IL-13 (100 or 200 µL of cell suspensions (35 × 10^3^ or 70 × 10^3^ MDM) were added to equal amount of MØ medium, containing 2× the desired final concentration of the cytokines). Macrophages were further incubated for 48 h before being assessed for fusion with the filovirus envelope (Env) pseudotyped, BLaM containing VLP. Based on previously published studies [48,49,50], the final cytokine concentrations were set at 20 ng/mL, unless otherwise indicated in the figure legends. There were 96 well black, clear plastic (Costar) or glass bottom (Greiner Bio) plates, suitable for fluorescent microscopy used for the experiments analyzed by Laser Scanning Cytometer. For the experiments analyzed by Flow cytometry, the cells were cultured in 48 well Nunc plastic plates. Infections were performed in triplicate as each well was evaluated by Laser Scanning Cytometry. Alternatively, the cells from the tree wells were combined in one tube before analysis by Flow Cytometry.

### 2.3. Cell Viability

Cell viability was evaluated using the Promega CellTiter-Glo^®^ Luminescent Cell Viability Assay, which is based on measuring the intracellular ATP levels as a marker of cellular metabolic activity.

### 2.4. BLaM Entry Assay

Filovirus GP pseudotyped, β-lactamase (BlaM)-containing virus particles were produced by co-transfection of 293T cells with plasmids encoding VP-40 BlaM, VP-40 and the relevant filovirus surface glycoprotein. The VLP-containing supernatants were collected after 24 and 48 h, subjected to low-speed centrifugation and low-protein binding 0.45 µm filtration to remove cell debris and then further concentrated by ultracentrifugation. The influenza HA and NA pseudotyped VLP were generated by co-transfecting 293T cells with plasmids encoding the relevant influenza proteins, the packaging plasmid psPAX2 and the Vpr-BlaM encoding plasmid pMM310. The HA/NA VLP were processed the same way as the filovirus VLP, except no further concentration by ultracentrifugation was necessary. The HA from the highly pathogenic A/Vietnam 2004 (H5N1) strain appears to be cleaved in the virus producing cells [51] and, therefore, does not require addition of exogenous trypsin or an alternative protease during the in vitro infection. The different filovirus VLP preps used in our studies were found to be relatively equivalent (based on number of particles per unit volume) by tests performed using NanoSight LM10 (Malvern Panalytical, Malvern, UK) and/or ViroCyt 3100 (ViroCyt/Sartorius, Boulder, CO, USA).

After infection for 3.5 h in DMEM, supplemented with L-glutamine, the cells were washed twice with phenol red and serum-free medium (DMEM with no phenol red, containing 2 mM L-glutamine, 25 mM HEPES, 2.5 mM of the nonspecific anion transport inhibitor probenecid) and loaded with the fluorescent dye CCF2/AM (2 μM final concentration in phenol red and serum free medium) for 1.5 h, washed twice with phenol red and serum free medium to remove the extracellular dye and incubated in phenol red free DMEM with 10% FCS, 2mM L-glutamine, 25mM HEPES and 2.5 mM probenecid for 12–14 h prior to fixing with 1.6% paraformaldehyde. The extent of CCF2/AM cleavage by the virus-introduced intracellular BlaM, detected by the change in dye emission from the green to the blue spectrum, was evaluated by measuring the fluorescence using Laser Scanning Cytometer (CompuCyte, Cambridge, MA, currently ThorLabs) or BD LSR II Cell Analyzer generating light at 407 nm and equipped with HQ460/10 and HQ530/20 filters for detection of the blue and green emission, respectively. Separate negative controls (mock infection) were prepared for both control and cytokine-treated cells but, in general, insignificant difference was observed in the capacity of the cells to uptake, retain and/or spontaneously cleave the CCF2/AM dye. The cells prepared for analysis by Laser Scanning Cytometry were also stained with the nuclear dye DRAQ5 (in combination with CCF2/AM) to improve the discrimination of individual cells, which were adjacent (in contact) with each other. On average, 5000 cells per sample were analyzed by Laser Scanning Cytometry and at least 10,000 cells per sample were analyzed by Flow cytometry.

The statistical analyses (*p* value calculations) were performed using GraphPad Prism software (unpaired two-tailed *t* test).

### 2.5. Flow Cytometry 

**A. Surface Staining:** MDM (10^6^ cells/sample) were pre-treated with IL-10 (20 ng/mL) or cytokine resuspension buffer in 6 well Nunc tissue culture plates for 48 h before being detached by pre-incubation in DPBS (without Ca^2+^ and Mg^2+^) at 4 °C and gentle scraping. The cells were washed once with DPBS supplemented with 3% bovine serum albumin (BSA) and incubated for 30 min at 4 °C with FcR blocking reagent (Miltenyi Biotec GmbH). Subsequently, the samples were incubated for additional 1.5 h at 4 °C with primary antibodies, washed 3 x times (DBPS plus 2% BSA) and either fixed in 1.6% paraformaldehyde (when fluorescently labeled primary antibodies were used), or further incubated with the relevant fluorophore conjugated secondary antibodies for 1 h at 4 °C, washed three times and fixed. Matching fluorescently labeled or unconjugated antibodies without specific binding activity were used as isotype controls. For the Niemann-Pick cholesterol transporter 1 (NPC-1) and cathepsin L labeling, the detached MDM were permeabilized and processed using the Foxp3/Transcription Factor Staining Buffer Set (Thermo Fisher Scientific), following manufacturer’s instructions with the addition of FcR blocking reagent incubation step. The fluorescent labeling of the viable cell populations (defined by FSC/SSC) was evaluated using BD LSR II or BD FACSCanto II cell analyzers. The data were processed and further analyzed using WinList software (Verity Software House, Topsham, MA, USA).

**B. EBOV GP binding studies:** The recombinant proteins wild-type receptor binding region (wtRBR) and the binding deficient 4merRBR, linked to rabbit IgG-Fc, and used to label cells of interest, were previously described [52,53]. In brief, wtRBR-Fc contains GP_1_ residues 57 to 149. The 4merRBR-Fc construct encodes mutations substituting the 4 lysine residues in GP_1_, found to be critical for RBR binding (K95A/K114A/K115A/K140A). Filovirus GP pseudotyped, green fluorescent protein (GFP)-containing virus particles were produced by co-transfection of 293T cells with plasmids encoding VP-40-GFP, VP-40 and the EBOV _Kikwit_ GP.The VLPs were concentrated and purified by ultracentrifugation through a sucrose cushion and were either kept frozen (−80 °C) or used immediately to label primary MDM for 2.5 h at 4 °C in a binding buffer (DPBS with Ca^2+^ and Mg^2+^ supplemented with 0.5% HSA), gently washed twice with ice cold binding buffer, fixed with paraformaldehyde and analyzed by flow cytometry (BD LSRII cell analyzer). To evaluate more accurately the EBOV GP binding we calculated and compared the staining indexes (SI) of the negative and positive pairs of mock and IL-10 treated MDM as previously described [54,55] (SI = mean _positive_ – mean _negative_/2 × SD_negative_)

### 2.6. Microarray Analysis

Primary MDM (3 × 10^6^ cells/sample) were mock treated (cytokine resuspension buffer – 0.5% HSA in DPBS) or pre-incubated with 20 ng/mL IL-10 for 48 h before cell mRNA was extracted and tested using the Qiagen RNeasy Kit and CS3011 Illumina Gene Exp. Beadchip, respectively. The microarray assay and analysis were performed at Qiagen (SABiosciences, Service Core for Gene Expression and Genomic Analysis, Frederick, MD).

### 2.7. Western Blot

In general, the samples were analyzed using the Invitrogen NuPAGE/Western blot system following manufacturer’s instructions. MDM (2 × 10^6^ cells/sample) were IL-10 or mock treated for 48 h before being detached (pre-incubation in DPBS without Ca^2+^ and Mg^2+^ at 4 °C and gentle scrapping) and lysed with RIPA lysis buffer plus protease inhibitors. 20 µL of each sample were combined with LDS (4X) concentrated sample buffer and 10 (x) concentrated reducing agent and loaded onto 4–12% Bis-Tris gels for electrophoresis. The samples were transferred to a nitrocellulose membrane, which was further developed using anti-cathepsin L (clone 33/1, Invitrogen) or anti-cathepsin B (clone 1J37, GeneTex) antibodies.

## 3. Results

### 3.1. IL-10, But Not TNF-α, IL-4 or IL-13, Increases Entry of EBOV GP_Kikwit_ Pseudotyped VLP in Primary Human MDM

**Figure 1:**
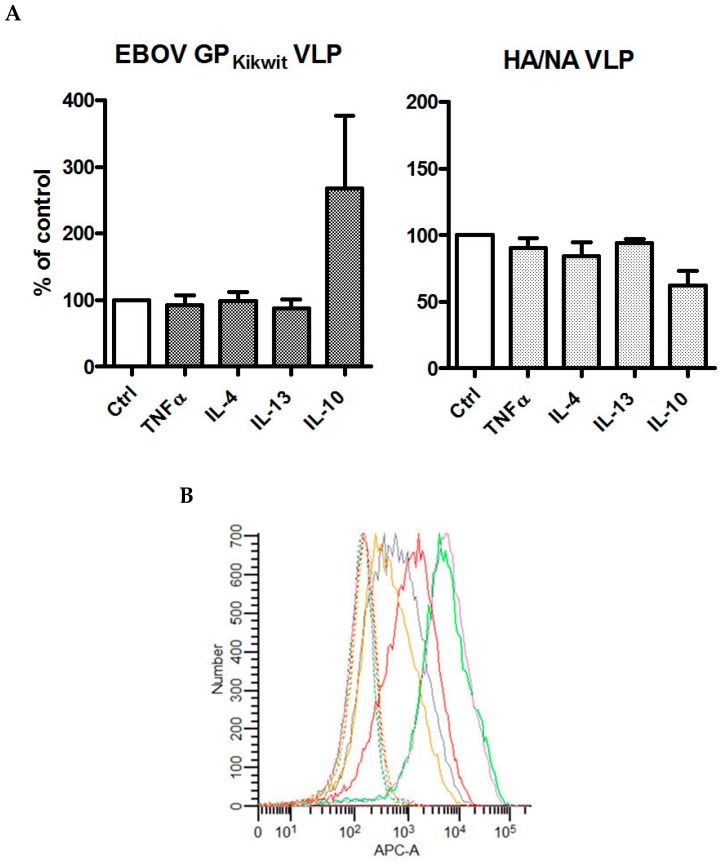
(**A**) IL-10, but not TNF-α, IL-4 or IL-13, significantly enhance EBOV_Kikwit_ glycoprotein(s) (GP) pseudotyped virus-like particles (VLP) entry in primary human monocyte-derived macrophages (MDM). MDM from three healthy donors were pre-treated with TNF-α, IL-4, IL-13 or IL10 (all at concentrations of 20 ng/mL) for 48 h prior to incubation with EBOV_Kikwit_ GP or influenza hemagglutinin/neuraminidase (HA/NA) pseudotyped VLP for 3.5 h at 37 °C. Subsequently, the cells were processed at room temperature, washed twice, loaded with CCF2/AM fluorescent dye for 1.5 h, washed again (2 times), incubated overnight to allow the cleavage of CCF2/AM by the VLP introduced β-lactamase (BlaM) and fixed in 1.6% paraformaldehyde. The extent of CCF2/AM cleavage, resulting in the change of the emission spectrum from 530 nm (green) to 460 nm (blue), was assessed by Laser Scanning Cytometry. The levels of VLP fusion with mock-treated MDM (treated with an equivalent volume of cytokine re-suspension buffer) were assumed to be 100%. Each treatment condition was performed in triplicate wells and the mean value(s) was used to calculate “% of control”. The data from the individual experiments, used to generate Figure 1A, are included in Table S1A through Table S1H (donor #1, donor #2 and donor #17). (**B**) TNF-α, IL-4, IL-13 or IL-10 affect CD206 expression in a cytokine-specific manner. Aliquots of MDM from one of the donors presented in Figure 1A were pre-incubated with TNF-α, IL-4, IL-13 or IL10 (20 ng/mL), detached by incubation in EDTA/EGTA buffer at 4°C and gentle scraping, and then labeled with phycoerythrin-conjugated anti-CD206 monoclonal antibody. CD206 cell surface expression was evaluated using BD FACSCanto II cell analyzer. The gray, yellow, red, green and purple lines represent control (mock treated), TNF-α, IL-10, IL-4 and/or IL-13 pre-incubated cells, respectively. The dash lines with the same colors represent the relevant isotype controls.

For our initial studies, we explored the effects of TNF-α, IL-4, IL-13 and IL-10 pre-incubation on the ability of primary human MDM from three healthy donors to support the fusion of VLP expressing EBOV GP_Kikwit_ or influenza HA and NA on their surface. Of the four cytokines tested, only IL-10 consistently enhanced EBOV GP_Kikwit_ VLP entry (Figure 1A). This is in agreement with a previous study [56] reporting that addition of IL-4 to macrophage colony stimulating factor (M-CSF) during monocyte/macrophage differentiation did not significantly affect EBOV VLP entry. The slight IL-10-induced reduction in HA/NA VLP entry (Figure 1A) also appears consistent with previous observations [57] regarding the effects of macrophage polarization on influenza infection of these cells.

Due to the high degree of variability in the published methods with respect to the concentrations of TNF-α, IL-4, IL-13 and/or IL10 (ranging from 5 ng/mL to 100 ng/mL) and/or the incubation times (from 4 to 175 h) used to elicit differential macrophage polarization [48,49,58,59,60,61,62,63,64,65,66], we sought to confirm that our experimental protocol induces the expected phenotypic changes. We chose a cytokine concentration (20 ng/mL) and an incubation time (48 h) close to the averages reported in the previous studies [48,49,59,63]. As a marker of cytokine effects [44,65], we used flow cytometry to assess CD206 cell surface expression on the MDM from one of the donors whose cells were also used in a virus entry/BlaM experiment summarized in Figure 1A. Consistent with published data regarding the CD206 expression on M2a, M2c or M1 macrophage phenotypes [50,62,65], CD206 was strongly upregulated by IL-4 and IL-13, increased to a lesser extent by IL-10, and slightly decreased by TNF-α, compared to the mock treated cells (Figure 1B). Because of the enhancing effect of IL-10 on EBOV GP_Kikwit_ VLP entry, we further characterized the IL-10 induced macrophage phenotype by flow cytometry and/or microarray analysis of control (mock treated with 0.5% HSA) and IL-10 (20 ng/mL) treated MDM from several additional healthy donors. The results from our microarray analysis (Supplemental Table S2) revealed a significant increase in Suppressor of Cytokine Signaling 3 (SOCS3), which is considered a consensus marker for IL-10 treated human macrophages [67]. We also confirmed the presence of increased levels of CD163 and IL21R by microarray analysis (Supplemental Table S1) and flow cytometry, previously reported to be upregulated by IL10 [58,65,68,69].

**Figure 2:**
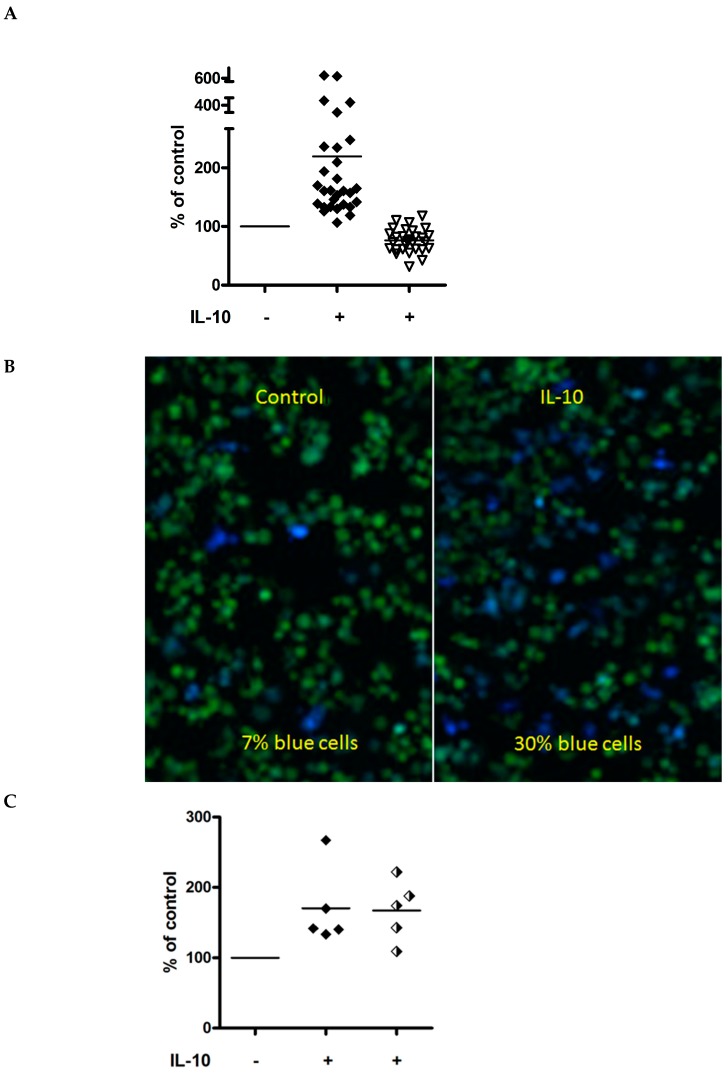

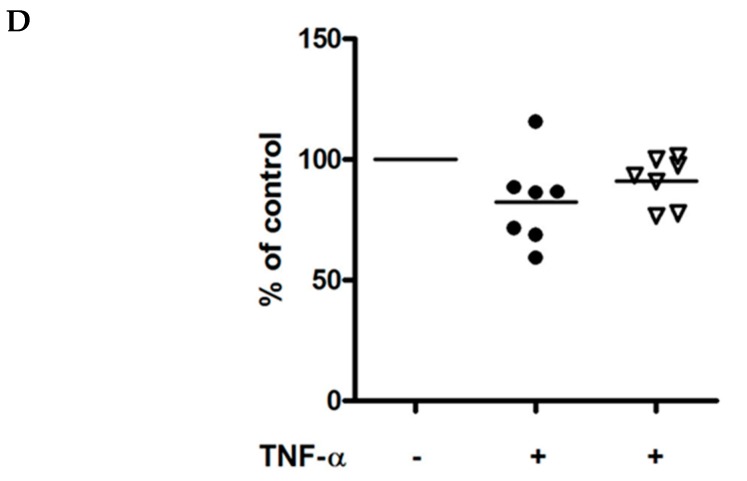
(**A**) IL-10 enhances entry of full-length EBOV_Kikwit_ GP pseudotyped VLPs in MDM from multiple donors (*n* = 25). MDM were pre-incubated with IL10 (20 ng/mL) for 48 h prior to infection. The cells were processed and the EBOV_Kikwit_ VLP (solid diamonds) entry/fusion was analyzed by Laser Scanning Cytometry as described in the Materials and Methods and Figure 1. BlaM-containing virus particles, pseudotyped with influenza HA/NA (white triangles), served as controls. The levels of virus entry in mock-treated MDM (with an equivalent volume of cytokine re-suspension buffer) were assumed to be 100%. The graph summarizes data from multiple experiments and includes the results for the IL-10 effect on EBOV_Kikwit_ VLP entry at a concentration of 20 ng/mL in the MDM of donors also used to generate the data presented in Figure 1, Figure 2B, Figure 3, Figure 4A–C, Supplemental Figure S1 and Supplemental Figure S2. The data, including statistical analysis, from the multiple experiments, used to generate Figure 2A, are summarized in Table S1A,B. (**B**) Representative fluorescent images, generated by the iCys Laser Scanning Cytometer using MDM from one of the donors, show increased numbers of cells fused with the EBOV GP VLPs (blue cells) after IL-10 treatment. No significant difference was observed in the background fluorescence of uninfected IL-10 or mock treated cells. (**C**) IL-10 induced enhancement is independent of the EBOV_Kikwit_ GP Δmucin domain. MDM from 4 healthy donors were infected in parallel with VLPs pseudotyped with either full length EBOV_Kikwit_ GP (solid diamonds) or EBOV_Kikwit_ Δ mucin GP (black and white diamonds). The cells were processed and the “% of control” was calculated as described for Figure 1. The data from the individual experiments, used to generate this figure, are included in Table S1A,B,I (donors #10, #11, #13, #15 and #29). (**D**) Donor-dependent effect of TNF-α on EBOV_Kikwit_ GP VLP entry into primary human MDM. In several different experiments, cells from a total of seven healthy donors were incubated with TNF-α (20 ng/mL) for 48 h, infected with EBOV_Kikwit_ GP VLP (solid circles) for 3.5 h, loaded with CCF2/AM fluorescent dye and processed as described in Materials and Methods. The subsequent evaluation of VLP fusion was performed either by Laser Scanning Cytometry (6 donors) or Flow cytometry (1 donor). The levels of VLP fusion with mock-treated MDM (equivalent volume of cytokine re-suspension buffer) were assumed to be 100%. VLPs pseudotyped with influenza HA/NA served as controls (open triangles). The data from the individual experiments, used to generate Figure 2D are summarized in Table S1C,F.

After the initially observed IL-10 enhancing effect on EBOV-GP_Kikwit_ VLP entry in several donors [70], we subsequently tested the effect of IL-10 in MDM obtained from more than 30 healthy individuals. Figure 2A summarizes the results from 25 donors, whose cells were analyzed using an iCys laser scanning cytometer. Figure 2B, shows fluorescent images of mock and IL-10 treated cells from one representative donor.

**Figure 3:**
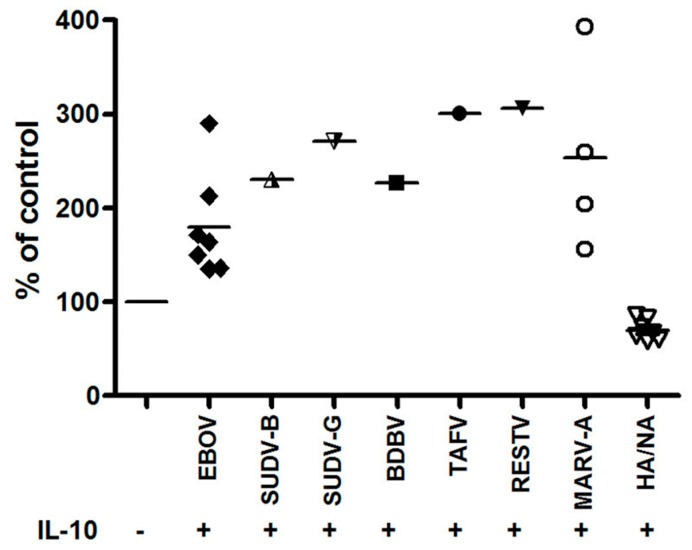
IL-10 enhances fusion of primary MDM with VLPs pseudotyped with envelope glycoproteins from all clinically significant filovirus species. Cells were pre-incubated with 20 ng/mL IL-10 or DPBS supplemented with 0.5% HSA (mock treated), infected, loaded with CCF2/AM, processed and analyzed by Flow cytometry as described in Materials and Methods. The figure summarizes the results from several different experiments, utilizing MDM from a total of seven different donors. In each experiment, MDM were infected with EBOV_Kikwit_ GP VLP (solid diamonds) in parallel with one or more different VLP types, pseudotyped with the surface glycoprotein of one of the indicated filovirus species. Data from one of the individual donors are presented in Supplemental Figure S3. Influenza HA/NA pseudotyped virus particles served as a control (open triangles).

MDM from seven additional donors were analyzed by flow cytometry for their ability to fuse with EBOV-GP_Kikwit_ VLP and/or VLP pseudotyped with the GP of alternative filovirus species (Figure 3), and the results were consistent with our observations by laser scanning cytometry. The effect of IL-10 (fold increase) on virus entry was more pronounced when the MDM were incubated with a reduced number of EBOV-GP_Kikwit_ VLPs (Supplemental Figure S1). We also compared the fusion of primary MDM with VLPs pseudotyped with full length EBOV-GP_Kikwit_ or Δ mucin EBOV-GP_Kikwit_ and observed comparable levels of IL-10 induced enhancement of VLP entry (Figure 2C), i.e., the effect of IL-10 does not appear to be dependent on the mucin domain of EBOV GP.

The enhancing effect of IL-10 was demonstrated as well for EBOV-GP_Kikwit_ VLP generated using the retrovirus-derived psPAX2 packaging plasmid and a HIV-1 Vpr-BLaM encoding construct (pVpr-Blam) (Supplemental Figure S2).

In parallel with IL-10, the effect of the pro-inflammatory cytokine TNF-α was tested in MDM isolated from 7 healthy donors (Figure 2D). Despite the observed donor to donor variability, in contrast to IL-10, the TNF-α pre-incubation displayed a tendency to slightly decrease EBOV_Kikwit_ VLP entry (in 6 of the 7 donors tested, Table S1C). TNF-α did not have a significant effect on HA/NA VLP entry into primary MDM (Table S1F).

### 3.2. IL-10 Augments the Macrophage Fusion with VLPs Pseudotyped with the Surface Glycoproteins of All Clinically Relevant Filovirus Species

Subsequent to testing the IL-10 effect on EBOV GP_Kikwit_ VLP entry, we demonstrated the ability of IL-10 to enhance the fusion of primary macrophages with VLP pseudotyped with the surface glycoproteins from most of the clinically relevant filovirus species: *Sudan ebolavirus,* strains Gulu and Boneface, *Taї Forest ebolavirus, Bundibugyo ebolavirus, Reston ebolavirus* and *Marburg Marburgvirus,* strain Angola. The data presented in Figure 3 were generated using MDM obtained from 7 healthy donors. The cells from each donor were infected in parallel with EBOV GP_Kikwit_ VLP and one or more additional VLPs, pseudotyped with one of the alternative filovirus glycoproteins (results from one representative donor are provided in Figure S3).

Although the level of IL-10 induced enhancement was similar across the different filovirus VLP tested, the data suggest that the VLPs pseudotyped with glycoproteins derived from more pathogenic species, such as EBOV, may fuse at a higher initial rate with primary human MDM (Figure S3).

### 3.3. IL-10 Enhances the Entry of EBOV GP VLPs Into MDM at Concentrations Observed In Vivo

**Figure 4:**
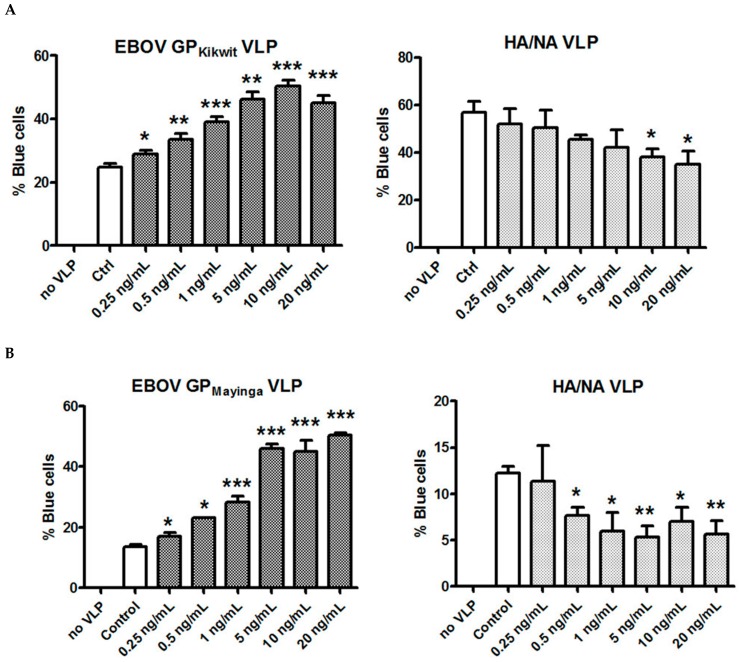

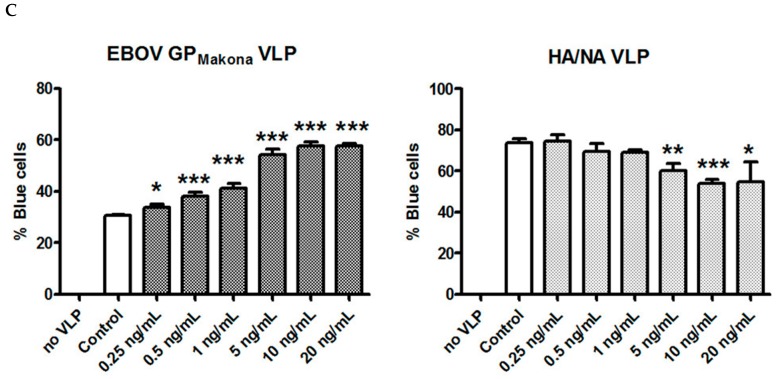
IL-10 enhances fusion of primary MDM with VLPs pseudotyped with EBOV GP in a dose-dependent manner, but has no significant effect or slightly inhibits entry of VLPs pseudotyped with influenza HA/NA. MDM from three different healthy donors were pre-incubated for 48 h with increasing concentrations of IL-10 and infected for 3.5 h with VLPs pseudotyped with the surface glycoproteins of the Kikwit (Panel **A**), Mayinga (Panel **B**) or Makona (Panel **C**) strains, respectively. Subsequently, the cells were washed, loaded with the fluorescent dye CCF2/AM and prepared for analysis by Laser Scanning Cytometry as described in Material and Methods. * *p* ≤ 0.05; ** *p* ≤ 0.01; *** *p* ≤ 0.001.

The IL-10 concentrations typically used to induce the M2c phenotype in vitro significantly exceed the IL-10 levels observed in vivo. Therefore, we tested the ability of IL-10 at concentrations ranging between 0.25 ng/mL and 20 ng/mL to affect the entry of VLPs pseudotyped with the surface glycoproteins of three EBOV isolates: Kikwit (Figure 4A), Mayinga (Figure 4B) and Makona (Figure 4C) into primary MDM.

We observed a significant, dose-dependent increase of entry for all three different types of VLPs, starting at the lowest IL-10 concentration of 0.25 ng/mL. Several previous studies have reported serum/plasma IL-10 levels in the 0.25 ng/mL-1 ng/mL range during EBOV infection, particularly in patients having an unfavorable outcome due to the disease [1,2,3,6,8,9] (see Supplemental Table S3 for more details). Furthermore, IL-10 levels up to 8 ng/mL have been detected during infection with SUDV [10]. It has also been established that EBOV GP and/or replication incompetent EBOV GP pseudotyped VLP are capable of inducing IL-10 secretion in cell culture models at concentrations between 0.025 and 5 ng/mL [16,17,18,26,27,28]. Because of the variety of methods and reagents used, it may be impossible to accurately compare the IL-10 levels established in different studies. However, taken in its entirety, the clinical and in vitro data support the concept that IL-10 levels higher than 0.25 ng/mL are very likely to be present in vivo at the sites of filovirus replication, given the in vitro established ability of filovirus surface glycoproteins to induce IL-10 secretion.

In contrast to our findings with EBOV GP pseudotyped VLPs, but consistent with our previous observations, IL-10 reduced the entry of influenza HA/NA VLPs into primary MDM in a dose-dependent manner (Figure 4 A, B, C).

### 3.4. The IL-10-Mediated Increase of EBOV-GP VLP Entry into Primary Human MDM May Occur at Binding and Potentially Post-Binding Steps

**Figure 5:**
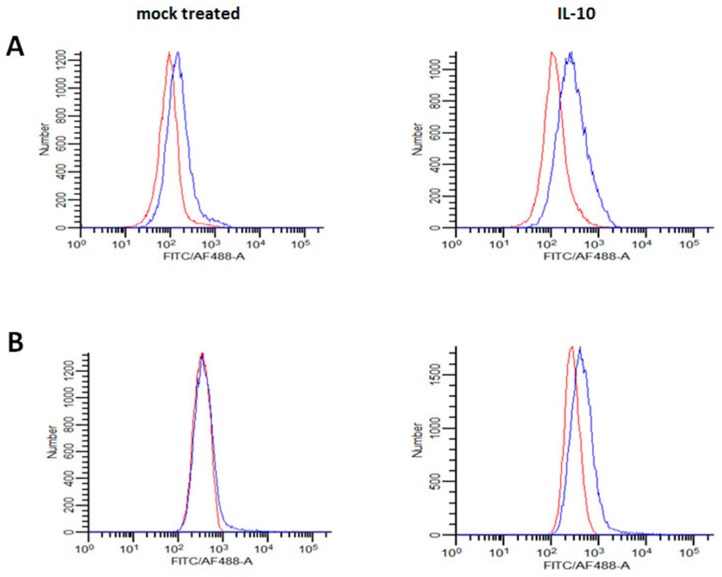
Increased binding of EBOV GP to IL-10 treated primary human MDM. (**A**) Cells were pre-treated for 48 h with IL-10 and incubated for 1.5 h at 4 °C in binding buffer (0.5% HSA in Ca^2+^ and Mg^2+^ containing DPBS) with a recombinant protein, consisting of the EBOV GP receptor binding domain (GP_1_ residues 57 to 149), fused to the Fc portion of rabbit IgG. MDM incubated with the binding deficient construct 4merRBR-Fc served as a negative control (red line). Subsequently, the MDM were washed, labeled with FITC conjugated polyclonal goat anti-rabbit antibody, fixed with paraformaldehyde and analyzed by Flow Cytometry. The staining indexes for the mock and IL-10 treated pairs of negative and positive samples are 0.3593 and 0.7086, respectively (for details regarding the staining indexes calculation please refer to Material and Methods). (**B**) Alternatively, MDM were incubated for 2.5 h at 4 °C with GFP-containing (VP40-GFP) VLPs pseudotyped with full length EBOV_Kikwit_ GP (blue line). Full length EBOV GP- pseudotyped VLPs, which do not contain GFP, served as a negative control (red line). The staining indexes for the mock and IL-10 treated pairs negative and positive samples are 0.8444 and 1.8228, respectively.

By using the EBOV GP-derived recombinant protein RBR-Fc [52,53] (Figure 5A) or GFP-containing VLPs (Figure 5B), we established by flow cytometry analysis that IL-10 pre-incubation increased EBOV GP binding to primary human MDM.

To further investigate the potential mechanism(s) responsible for the IL-10-mediated enhanced EBOV GP pseudotyped VLP entry, we performed a microarray analysis of IL-10 and/or mock treated MDM. The microarray study (Supplemental Table S2) did not clearly define a factor(s) responsible for the increased EBOV GP binding but suggested that IL-10 may also affect filovirus entry at both binding and post-binding steps. Of the molecules previously implicated in playing a role in filovirus cellular entry [lectins, TAM receptor tyrosine kinases (Tyro3, Axl, Mer), TAM receptor tyrosine kinases ligands (Protein S), T cell immunoglobulin and mucin domain (TIM) proteins, Toll-like receptors, integrins (αV and β1), cathepsins (L and B) and Niemann-Pick cholesterol transporter 1 (NPC1)] (reviewed in [71,72,73]), we found increased mRNA expression of integrin αV, cathepsin L, Protein S and Axl. Subsequently, we confirmed by immuno-fluorescence the upregulation of integrin αV/CD61 and Axl. Integrin β1/CD29 mRNA was not found to be increased using the microarray testing, but the MDM cell surface expression (assessed by flow cytometry) was slightly increased, most likely as a result of its association with αV/CD61 (data not shown). The microarray analysis did not find upregulation of TIM proteins, but revealed upregulated Protein S mRNA. Although largely unstudied for its potential role in virus entry, Protein S has the capability to bridge phosphatidyl serine (via its N-terminal domain) of the virus membrane to the TAM receptors on the cell surface [74]. Cathepsin L, which is involved in the partial endosomal cleavage of EBOV GP to its fusion competent form, showed significant donor to donor variability (being upregulated by IL-10 in some, but not all, donors), when tested by flow cytometry and/or western blot (Supplemental Figure S4 and data not shown). No upregulation of NPC-1 was detected in primary MDM by microarray analysis or flow cytometry (data not shown).

### 3.5. The Enhancing Effect of IL-10 on EBOV GP_Kikwit_ -Mediated Fusion/VLP Entry in Primary Human MDM Is Preserved in the Presence of Pro-Inflammatory Cytokines

**Figure 6:**
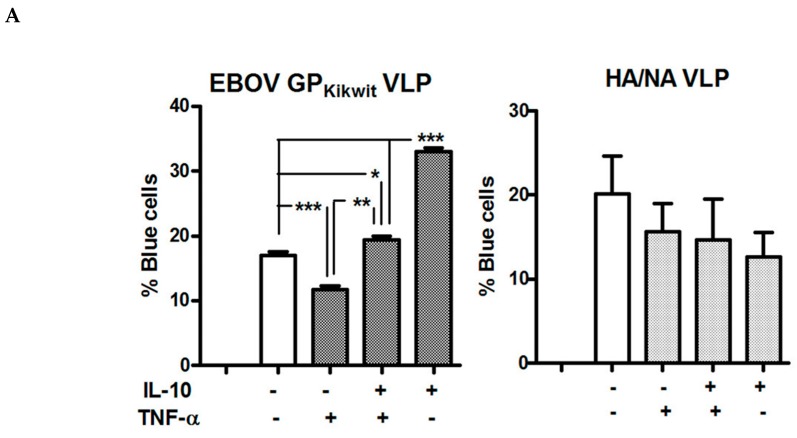

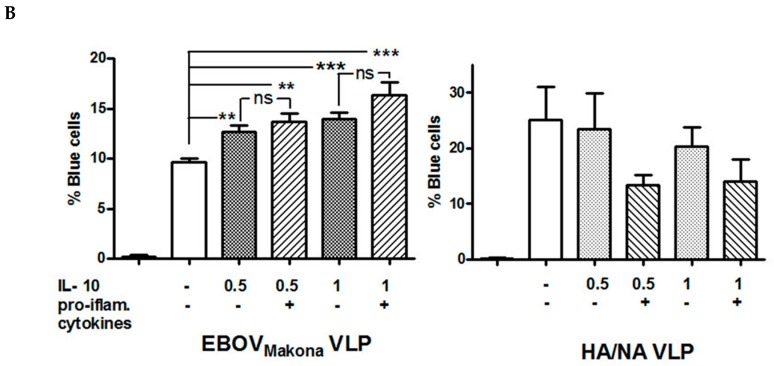
The enhancing effect of IL-10 on EBOV VLP entry is preserved in the presence of pro-inflammatory cytokines. (**A**) MDM were pre-treated with IL-10, TNF-α or a combination of both for 48 h (IL-10 and TNF-α were used at a concentration of 20 ng/mL) and infected with EBOV_Kikwit_ GP VLP. The first columns from the left represent mock infected cells. There was no difference in the background fluorescence of mock-infected MDM incubated with cytokines or in medium alone. (**B**) MDM were pre-incubated with IL-10 (0.5 ng/mL or 1 ng/mL) or IL-10 plus a cocktail of pro-inflammatory cytokines (TNF-α, 0.1ng/mL; MIP-1α, 0.1ng/mL; MIP-1β and MCP-1, 2ng/mL; IL-8, 0.5ng/mL) for 48 h before infection. After the 3.5 h infection period, the cells were prepared for analysis by Laser Scanning Cytometry as described in Materials and Methods. * *p* ≤ 0.05; ** *p* ≤ 0.01; *** *p* ≤ 0.001; ns = not significant.

In general, EBOV infection is characterized as having concomitantly increased levels of IL-10 and pro-inflammatory cytokines [1,2,3,8,9,33]. Initially, we investigated the IL-10 effect on EBOV_Kikwit_ VLP entry (Figure 6A and Table S1C) in the presence of TNF-α at cytokine concentrations (20 ng/mL) usually applied to induce macrophage phenotype changes in vitro (see Materials &Methods). Using MDM isolated from two healthy donors, we observed a modest reduction of EBOV_Kikwit_ VLP entry following TNF-α pre-incubation, while IL-10 counteracted this effect (Figure 6A and Table S1C, MDM from one of the donors were analyzed using Laser scanning cytometry, while the cells from the other donor were analyzed by Flow cytometry). Although the differences were not statistically significant, both IL-10 and TNF-α slightly decreased the entry of HA/NA VLP in the primary MDM of these donors. The TNF-α-induced reduction in EBOV_Kikwit_ VLP fusion appears consistent with previous findings [2,4,5] showing that a strong pro-inflammatory cytokine response, including elevated TNF-α secretion, may be protective, at least in some donors, early in EBOV infection. Subsequently, we tested in parallel the effects on EBOV_Makona_ VLP entry in the presence of low concentrations of IL-10, potentially present during EBOV infection, with IL-10 applied alone or in combination with other cytokines, modeled after the cytokine profile characterized in non-survivors during the recent West Africa outbreak [3] and again observed increased VLP-MDM fusion in both cases (Figure 6B). The same combinations of IL-10 and pro-inflammatory cytokines had either no significant effect or decreased the entry of influenza HA/NA pseudotyped VLP in primary human MDM. Intriguingly, unlike high levels of TNF-α (Figure 6A), the pro-inflammatory cytokine cocktails (Figure 6B) do not show a tendency to counteract the enhancing effect of IL-10 on EBOV GP VLP entry. A unique signaling pattern induced by the cytokine cocktail, compared to TNF-α alone, may be one of the potential factors responsible for the observed difference. Studies are ongoing to further investigate this initial observation.

## 4. Discussion

Along with higher virus loads, increased IL-10 levels have consistently emerged as a prognostic factor for poor clinical outcomes in EBOV [1,2,3,8,9,33], SUDV [10] and/or BDBV [11] infection (see Supplemental Table S2 for details).

There is also evidence that the dynamics of IL-10 changes may be associated with filovirus pathogenicity. A comparison between an eight day long in vitro infection of human PBMCs with EBOV (Mayinga strain) and BDBV, which is associated with lower mortality rate, established higher IL-10 levels in the supernatants of EBOV infected cells up to day 5 post-infection with a peak on day 2, while the IL-10 concentration became higher in BDBV supernatants on days 6 and 8 post infection [13]. This observation appears consistent with a subsequent study which established higher IL-10 levels in fatal cases of BDBV infection in serum samples with a median time of collection 7 (non-survivors) to 7.5 days (survivors) post onset of illness [11]. Although a comparison among clinical studies should be made with caution because of differences in the study designs and methodologies, there are data suggesting that in EBOV [1,2] and/or SUDV [10,75] infections with high mortality rates, IL-10 is markedly elevated within the first 4–5 days after the onset of symptoms. No clear pattern has been established for the 2014–2016 Makona EBOV outbreak, which had a relatively lower mortality rate (~40–50%) [76,77,78] compared to previous EBOV outbreaks [78,79,80]. Increased serum/plasma IL-10 levels in non-survivors, compared to survivors, were detected before day 7 of onset of symptoms in patients from Guinea [3,9,81], but significantly higher IL-10 levels in fatal cases were detected only 7 days after symptom manifestation in serum samples collected from patients in Sierra Leone [33]. Intriguingly, the WHO Ebola Situation Report from 30 March, 2016, indicated a higher mortality rate (reported deaths among probable and suspected cases vs. all confirmed, probable, and suspected cases) in Guinea [(2543/3811) × 100 = 66.7%] compared to Sierra Leone [(3956/14124) × 100 =28%]. Whether distinct IL-10 responses were a contributing factor for the different mortality rates in Guinea and Sierra Leone during the Makona outbreak remains to be established.

IL-10 changes have been shown to play a role not only in humans, but also in other species susceptible to filovirus infection:

Elevated IL-10 serum concentrations were detected in Cynomolgus macaques (over 10 ng/mL in some animals) infected with RESV [82]. Increased IL-10 plasma/serum levels were also observed in EBOV infected Cynomolgus macaques [76,83], but no significant changes in IL-10 were established in other studies using the same animal model [84,85]. Interestingly, no changes in IL-10 were reported when rhesus macaques were challenged intramuscularly (IM) with MARV-Angola [86], but multiple-fold increases in serum IL-10 levels were established in another study after aerosol exposure of rhesus macaques to the same virus [87]. These results suggest that the route of filovirus exposure may affect the changes in IL-10 levels during the subsequent infection and raise the question of whether the typical IM challenge of non-human primates (NHP) may be a factor for the less frequently observed IL-10 increase in these animals, compared to the naturally occurring filovirus infections in humans.

In addition to being a natural, usually asymptomatic host of RESV [88,89], domestic pigs are susceptible to and develop severe respiratory pathology in response to EBOV infection (after a combined intranasal, intraocular and oral challenge), which was associated with up to a 20-fold increase of IL-10 mRNA in the lung tissue of the infected animals [90,91].

Increased IL-10 levels were also found in the mouse model of EBOV infection [29,30,31,92]. The significance of IL-10 signaling in the pathogenesis of EBOV was further demonstrated [93] by showing that IL10^-/-^ knock-out mice or mice treated with IL-10 specific anti-sense phosphorodiamidate morpholino oligomers (PMOs) had significantly lower virus titers and higher survival rate (70% vs. 30%) after intraperitoneal challenge with mouse adapted EBOV. Specific antibody-mediated depletion experiments implied that NK cells and interferon-γ (IFN-γ) are required for the protection of IL10^-/-^ knock-out mice, leading to the hypothesis that IL-10 may aggravate EBOV infection via suppression of the immune system [93].

Our results indicate that, in addition to these general effects on the immune system, IL-10 can specifically influence filovirus infection by enhancing virus entry in primary human macrophages. After our initial observations [70], we consistently observed the enhancing effect of IL-10 on EBOV GP_Kikwit_ VLP entry in MDM isolated from more than 30 healthy donors (Figure 2A and Figure 3). We subsequently established that the IL-10 effect on VLP entry is likely mediated at cell binding and post-binding steps (Figure 5). In principle, a scenario whereby higher IL-10 levels augment the ability of macrophages to support filovirus fusion appears consistent with a proposed mathematical model of EBOV disease suggesting that the higher viral load in non-survivors is associated with an accelerated conversion of “potential target cells” into “susceptible target cells” during infection [94].

We observed that IL-10 treatment increased the entry into primary human MDM of VLPs pseudotyped with the surface GP from all filovirus species tested, regardless of their in vivo pathogenicity, to a similar extent (Figure 3). However, it was previously established that the IL-10 production during infection may correlate with the differing filovirus pathogenicity in a particular host species. Exposure of human PBMC to a 17 amino acid (AA) peptide derived from EBOV GP induced IL-10 secretion, in contrast to an analogous peptide from RESV, asymptomatic in humans, which did not [27].

Importantly, we demonstrated that for all three well-studied EBOV strains (Mayinga, Kikwit and Makona), the IL-10 enhancing effect on virus entry occurs not only at concentrations used to alter the macrophage phenotype in vitro, but also at considerably lower levels, which are likely to be present during filovirus infection (Figure 4 A–C and Supplemental Table S3). More importantly, the enhancing effect of IL-10 on EBOV VLP fusion was preserved in the presence of pro-inflammatory cytokines (Figure 6A and Figure 6B) shown to be elevated during EBOV infection [3].

The potential impact of macrophage polarization on filovirus pathogenesis is supported by two recent studies. Using a humanized mouse model, Lavender et al. [95] demonstrated that EBOV infection in these animals is associated with a higher frequency of terminally differentiated “M2-like” CD14^+^/CD163^+^ macrophages. MARV, shown to be less pathogenic in this particular animal model, caused “a more balanced activation of the M1-like and M2-like macrophage subsets”. In the second study, McElroy et al. [96] established that high levels of soluble CD163 and/or increased expression of cell-surface CD163 on tissue macrophages is associated with increased mortality or severity of SUDV or EBOV infection, respectively. It was hypothesized that macrophage activation contributes to the pathogenesis of filovirus infection and an analogy was made with the macrophage activation syndrome and the hemophagocytic lympho-histiocytosis. In support of this model, a reference was made to a previous finding that CD163 may be expressed on both M1 and M2 macrophages [97], but a more detailed characterization of the macrophage phenotype was not performed on the tissue samples obtained from the filovirus infected patients [96]. On the other hand, IL-10, which is associated with increased severity/mortality of filovirus infection, is also a known inducer of CD163 expression ([58,65,68] and our own results) and macrophage polarization to the M2c phenotype.

**Figure 7:**
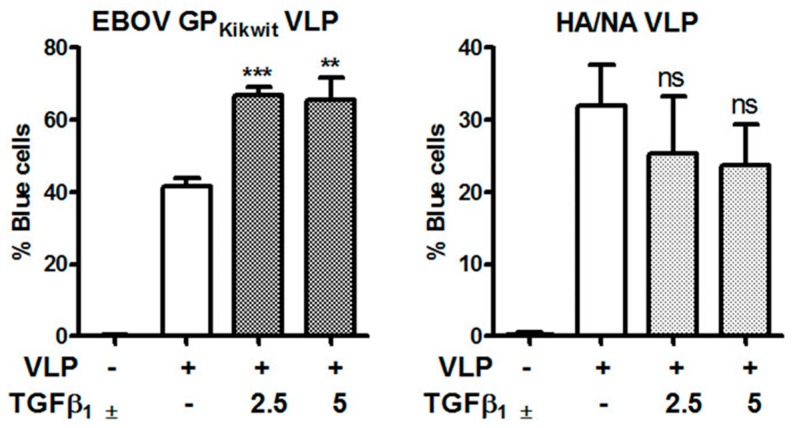
TGF-β1 enhances fusion of primary MDM with VLPs pseudotyped with EBOV_Kikwit_ GP. MDM were pre-incubated for 72 h in macrophage medium supplemented with TGF-β1 (2.5 or 5 ng/mL) or cytokine resuspension buffer (0.5% HSA DPBS) prior to infection with EBOV_Kikwit_ VLP or HA/NA VLP, respectively. The cells were processed and the VLP entry/fusion was analyzed by Laser Scanning Cytometry, as described in Materials and Methods. No significant difference was observed in the background fluorescence of uninfected TGF-β1 or mock treated cells. ** *p* ≤ 0.01; *** *p* ≤ 0.001; ns = not significant.

Indeed, in a recent review discussing the role of mononuclear phagocytes in EBOV infection [98], it was speculated that M2 macrophage polarization may enhance EBOV infection. Our study, looking solely at the level of virus entry, has demonstrated that M2c, but not the M2a macrophages, are more prone to fusion with filovirus GP pseudotyped VLP. Whether M2 macrophages, compared to the M1 phenotype, are in general more susceptible to EBOV infection at post-entry steps remains to be determined. We also generated preliminary data, showing that MDM treatment with TGF-β_1_, another cytokine linked to the M2c-like phenotype (reviewed in [99,100,101]), is also capable of enhancing EBOV VLP fusion (Figure 7). However, further studies are needed to determine if the observed enhancing effect of IL-10 and/or TGF-β_1_ on VLP entry is mediated by the same factors and to delineate whether TGF-β_1_ affects EBOV VLP fusion directly or via potential TGF-β_1_ induced IL-10 secretion [102,103].

Intriguingly, the findings that pre-incubation of primary MDM with IL-10 in combination with several pro-inflammatory cytokines enhances filovirus VLP entry, compared to mock treated controls (Figure 6A,B), imply that the increased ability of macrophages to support filovirus fusion may not be uniquely associated with the typical in vivo M2c phenotype, which is usually induced by adding just IL-10 to the cell culture medium. Whether the same set of molecules is involved in augmenting VLP entry in macrophages exposed to IL-10 alone or IL-10 in combination with pro-inflammatory cytokines is currently being investigated.

It is important to know if changes in IL-10, induced by a concomitant infection, may influence the clinical outcome of EVD. During the 2014–2016 EBOV outbreak, both increased [104,105] or decreased [106,107] survival rates were reported in patients co-infected with malaria species. Malaria is known to exert variable effects on IL-10, depending on the severity of infection (lower IL-10 in complicated cases, compared to asymptomatic subjects [108,109,110,111]), but cytokine levels, including IL-10, have not been evaluated in the currently published studies on EBOV and malaria co-infection.

In general, IL-10 exerts a pleiotropic effect to ensure a balance between pro- and anti-inflammatory immune responses in the context of a changing host environment during infection. A variety of pathogens have developed different mechanisms to take advantage of the immuno-suppressive effects of IL-10 and certain viruses have the ability to either augment host IL-10 production or encode biologically active IL-10 homologs, as a way to delay virus clearance and/or establish a persistent infection (reviewed in [112,113,114,115,116]. Our results demonstrate that filoviruses may not only employ the general IL-10 effects on the innate and adaptive immune responses [93,117], but may also take advantage of the IL-10 ability to specifically influence macrophage polarization towards a phenotype which favors filovirus fusion and entry, thus emphasizing the need to better understand the complex role of IL-10 in filovirus pathogenicity, as a way to provide new insights for improving treatment and reducing mortality of filovirus infection. Perhaps most importantly, in a setting of limited resources, our findings here suggest a path forward to extend the array of biomarkers of disease progression, to facilitate the adequate identification of patients in greatest need of medical attention.

## Supplementary Materials

The following are available online at https://www.mdpi.com/1999-4915/11/10/889/s1, **Figure S1**: IL-10 has a more pronounced enhancing effect (fold increase) when monocyte-derived macrophages (MDM) are infected with a reduced amount of EBOV_Kikwit_ virus-like particles (VLP); **Figure S2**: The IL-10 enhancing effect is independent of the type of packaging plasmid used to generate the EBOV_Kikwit_ GP pseudotyped VLPs.; **Figure S3**: IL-10 enhances fusion of primary MDM with VLPs pseudotyped with envelope glycoproteins from different filovirus species.; **Figure S4**: Effect of IL-10 on Cathepsin L expression.; **Table S1A**: Effect of IL-10 on VLP GP_Kikwit_ VP40-BlaM entry into primary MDM; **Table S1B**: Effect of IL-10 on VLP HA/NA Vpr-BlaM entry into primary MDM; **Table S1C**: Effect of TNF-α on VLP GPKikwit VP40-BlaM entry into primary MDM; **Table S1D**: Effect of IL-4 on VLP GPKikwit VP40-BlaM entry into primary MDM; **Table S1E**: Effect of IL-13 on VLP GPKikwit VP40-BlaM entry into primary MDM; **Table S1F**: Effect of TNF-α on VLP HA/NA Vpr-BlaM entry into primary MDM; **Table S1G**: Effect of IL-4 on VLP HA/NA Vpr-BlaM entry into primary MDM; **Table S1H**: Effect of IL-13 on VLP HA/NA Vpr-BlaM entry into primary MDM; **Table S1I**: Effect of IL-10 on VLP Δ mucin GPKikwit VP40-BlaM entry into primary MDM; **Table S1J**: Effect of IL-10 on VLP GPKikwit Vpr-BlaM entry into primary MDM; **Table S2**: Significant changes in IL-10-induced gene expression of selected molecules associated with M2c macrophage polarization or EBOV cellular entry.; **Table S3**: Changes in IL-10 levels observed in survivors and non-survivors during filovirus infection.
