# Supplementary material for: Cytokine Effects on the Entry of Filovirus Envelope Pseudotyped Virus-Like Particles into Primary Human Macrophages
Source: Viruses. 2019 Sep 23;11(10):889. doi: 10.3390/v11100889 (PMC6832363; doi:10.3390/v11100889)
Supplement: Supplementary file 1 [file viruses-11-00889-s001.pdf]

Supplementary Materials

Table S1A

Effect of IL-10 on virus-like particles (VLP) GP_{Kikwit} VP40-BlaM entry into primary MDM

Donor #	Control (0.5% HSA) % Blue cells Mean ± SEM	IL-10 20 ng/mL % Blue cells Mean ± SEM	*p* value	% Control
1	18.5 ± 0.89	28.3 ± 1.3	0.0004***	153.1
2	14 ± 1.0	22.5 ± 1.5	0.0084**	160.7
3	35 ± 4.6	41.7 ± 2.65	0.0751 ns	119
4	33.3 ± 1.86	43.3 ± 1.33	0.0119*	130
5	24.7 ± 0.33	40.7 ± 1.33	0.0003***	164.9
6	58.17 ± 3.35	62 ± 1.16	0.4652 ns	106.6
7	19.3 ± 0.88	25.7 ± 1.2	0.0041**	132.8
8	16 ± 3.46	22 ± 3.61	0.2964 ns	137.5
9	44 ± 1.53	58.7 ± 0.88	0.0011**	133.4
10	16.75 ± 1.44	23.25 ± 1.44	0.0186*	138.8
11	10 ± 0.58	26.67 ± 2.67	0.0036**	267
12	43.8 ± 1.58	64 ± 0	0.0067**	146
13	22 ± 1.51	29.33 ± 0.88	0.0147*	133.3
14	21.67 ± 0.99	35 ± 2.89	0.0008***	161.5
15	14.3 ± 4.37	20.3 ± 2.33	0.2926 ns	141.9
16	6.33 ± 0.88	15.67 ± 1.45	0.0054**	247.6
17	4.2 ± 0.4	20.3 ± 2.85	<0.0001***	483.3
18	17 ± 0.58	33 ± 0.58	<0.0001***	194.1
19	8.83 ± 0.4	57.67 ± 2.96	<0.0001***	653.1
20	17.17 ± 0.91	27 ± 0.58	0.0002***	157.3
21	7.33 ± 2.03	17.33 ± 1.67	0.0189*	236.4
22	24.83 ± 1.08	45 ± 2.52	<0.0001***	181.2
23	28.33 ± 1.5	45.5 ± 2.5	0.0012**	160.6
24	10.33 ± 0.33	21.67 ± 1.86	0.0039**	209.8
25	8.67 ± 0.67	20.33 ± 1.45	0.0019**	234.5
26	6.67 ± 0.88	30 ± 1.56	<0.0001***	449.8
27	6.33 ± 0.88	40.33 ± 0.33	<0.0001***	637.1
28	56 ± 0.58	70.67 ± 0.67	<0.0001***	126.2
29	37.67 ± 0.33	64 ± 4.36	0.0038**	169.9

HSA: human serum albumin; SEM: standard error of the mean

The *p* values were calculated by GraphPad Prism software using the unpaired, two tailed t-test method. * $p \leq 0.05$; ** $p \leq 0.01$; *** $p \leq 0.001$; ns = not significant.

"% of Control" is used as an equivalent of "Fold increase" (% of Control/100 = Fold increase).

The % blue cells in the mock infected samples is <1%.

In general, the donors' numbers in the table(s) reflect the chronology of the experiments and cannot be used for personal identification.

Table S1B

Effect of IL-10 on VLP HA/NA Vpr-BlaM entry into primary MDM

Donor #	Control (0.5% HAS) Mean ± SEM	IL-10 20 ng/mL Mean ± SEM	*p* value	% control
1	78.5 ± 1.84	63.3 ± 7.54	0.0307*	80.7
2	52.3 ± 2.78	33 ± 5	0.0205*	63.2
3	56 ± 3.06	34 ± 10.5	0.1154 ns	60.7
4	21.3 ± 2.4	11.7 ± 1.67	0.0298*	54.7
5	20.3 ± 4.84	24 ± 4.58	0.6116 ns	118.1
6	40.7 ± 2.36	33.7 ± 3.33	0.1306 ns	82.8
7	23.2 ± 1.3	18 ± 2	0.0605 ns	77.7
8	29 ± 1.0	32 ± 1.0	0.1377 ns	110.3
9	65.7 ± 9.26	55.7 ± 2.6	0.3573 ns	84.8
10	51 ± 6.62	37 ± 4.02	0.1207 ns	72.5
11	22.67 ± 3.28	21 ± 4.51	0.7800 ns	92.6
12	70 ± 4.32	42.3 ± 2.03	0.0036**	60.5
13	24.17 ± 4.41	7.67 ± 1.33	0.0390*	31.7
14	32.33 ± 3.87	17.33 ± 0.33	0.0331*	53.6
15	4.33 ± 1.33	2.67 ± 0.67	0.3262 ns	61.7
16	11 ± 0.58	9 ± 0.58	0.0705 ns	81.8
17	37.8 ± 3.83	16 ± 1.73	0.0066**	42.3
18	20,17 ± 4.43	12.67 ± 2.91	0.3038 ns	62.9
19	41.17 ± 5.04	30 ± 6	0.2235 ns	72.9
20	81.67 ± 6.38	79.67 ± 3.38	0.8409 ns	97.6
21	14.67 ± 3.18	11 ± 0.58	0.3199 ns	75
22	56.67 ± 4.86	35 ± 5.57	0.0301*	61.8
23	79.17 ± 2.8	77 ± 3	0.6969 ns	97.3
24	44 ± 3.22	47 ± 6.66	0.7057 ns	106.8
25	71.67 ± 4.49	68.33 ± 1.76	0.5272 ns	94.8
26	23 ± 4.51	15.67 ± 1.45	0.1966 ns	68.1
27	35.67 ± 5.46	29.67 ± 3.93	0.4227 ns	83.2
28	32 ± 11.53	23.67 ± 2.33	0.5179 ns	74
29	52.33 ± 1.45	46 ± 0.58	0.0155*	87.9

Table S1C

Effect of TNF-*α* on VLP GP_{Kikwit} VP40-BlaM entry into primary MDM

Donor #	Control (0.5% HAS) % Blue cells Mean ± SEM	TNF-*α* 20 ng/mL % Blue cells Mean ± SEM	*p* value	% Control
1	18.5 ± 0.89	16.00 ± 0.58	0.1064 ns	86.5
2	14 ± 1.0	10 (10 ± 0.05)	0.058 ns	71.4
13	22 ± 1.51	19 ± 1.73	0.2654 ns	86.4
15	14.33 ± 4.37	12.67 ± 2.19	0.7503 ns	88.4

17	4.2 ± 0.4	5 ± 0.58	0.2718 ns	119
18	17 ± 0.58	11.67 ± 0.67	0.0008***	68.6*
	48.7	28.9	NA	59.3 ^

* The results from donor #18 are also presented in Figure 6A.

^ The MDM from the last donor were tested by Flow cytometry in triplicate. The cells from each of the 3 well sets were detached and combined in one tube before Flow cytometry analysis and, therefore, SEM and *p* value were not calculated (NA-not applicable). The levels of VLP entry into MDM pre-incubated with IL-10 alone or IL-10 plus TNF-α (concentrations of 20 ng/mL) are 56.4% and 32.7% blue cells, respectively.

Table S1D

Effect of IL-4 on VLP GP$_{\text{Kikwit}}$ VP40-BlaM entry into primary MDM

Donor #	Control (0.5% HAS) Mean ± SEM	IL-4 20 ng/mL Mean ± SEM	*p* value	% Control
1	18.5 ± 0.89	15.00	0.0358*	81.1
2	14 ± 1.0	17.5 ± 5	0.0842 ns	125
17	4.2 ± 0.4	3.7	0.6682 ns	88.1

Table S1E

Effect of IL-13 on VLP GP$_{\text{Kikwit}}$ VP40-BlaM entry into primary MDM

Donor #	Control (0.5% HAS) Mean ± SEM	IL-13 20 ng/mL Mean ± SEM	*p* value	% Control
1	18.5 ± 0.885	16.33 ± 1.86	0.2620 ns	88.1
2	14 ± 1.0	15.5 ± 0.5	0.3827 ns	110.7
17	4.2 ± 0.4	2.7 ± 0.67	0.0796 ns	63.5

Table S1F

Effect of TNF-α on VLP HA/NA Vpr-BlaM entry into primary MDM

Donor #	Control (0.5% HAS) Mean ± SEM	TNF-α 20 ng/mL Mean ± SEM	*p* value	% Control
1	78.5 ± 1.84	73.33 ± 3.84	0.2037 ns	93.4
2	52.3 ± 2.78	53 ± 0.05	0.8572 ns	101.3
13	24.17 ± 4.41	22 ± 1.73	0.7505 ns	91
15	4.33 ± 1.33	4.33 ± 0.33	1.0 ns	100
17	37.8 ± 3.83	29 ± 2.52	0.1747 ns	76.7
18	20.17 ± 4.3	15.67 ± 3.33	0.5318 ns	77.7
	87.6	85.6	NA	97.7^

^The levels of HA/NA VLP entry into MDM pre-incubated with IL-10 alone or IL-10 plus TNF-α (concentrations of 20 ng/mL) are 83.8% blue cells, respectively.

Table S1G

Effect of IL-4 on VLP HA/NA Vpr-BlaM entry into primary MDM

Donor #	Control (0.5% HAS) Mean ± SEM	IL-4 20 ng/mL Mean ± SEM	*p* value	% Control
1	78.5 ± 1.84	79.33 ± 2.96	0.8089 ns	101.1
2	52.3 ± 2.78	34 ± 9	0.0575 ns	65
17	37.8 ± 3.83	33 ± 1,16	0.4216	87.3

Table S1H

Effect of IL-13 on VLP HA/NA Vpr-BlaM entry into primary MDM

Donor #	Control (0.5% HAS) Mean ± SEM	IL-13 20 ng/mL Mean ± SEM	*p* value	% Control
1	78.5 ± 1.84	78.67	0.9724 ns	100.5
2	52.3 ± 2.78	46.5 ± 3.5	0.2873 ns	88.9
17	37.8 ± 3.83	35.33	0.6910 ns	93.5

Table S1I

Effect of IL-10 on VLP Δ mucin GP_{Kikwit} VP40-BlaM entry into primary MDM

Donor #	Control (0.5% HAS) Mean ± SEM	IL-10 20 ng/mL Mean ± SEM	*p* value	% Control
10	17.5 ± 1.76	25 ± 2.48	0.0487*	142.9
11	9 ± 1.16	15.67 ± 2.96	0.1041 ns	174.1
13	29.3 ± 2.58	32 ± 3.06	0.5521 ns	109.1
15	10.7 ± 0.67	23.7 ± 4.06	0.0341*	221.8
29	16.33 ± 1.76	30.67 ± 2.6	0.0104*	187.8

Table S1J

Effect of IL-10 on VLP GP_{Kikwit} Vpr-BlaM entry into primary MDM

Donor #	Control (0.5% HAS) Mean ± SEM	IL-10 20 ng/mL Mean ± SEM	*p* value	% Control
8	36.33 ± 3.28	68 ± 2	0.0012**	187.2
24	31.33 ± 1.33	44.33 ± 1.45	0.0027**	141.5
25	27.67 ± 1.2	49 ± 0.58	<0.0001***	166.2

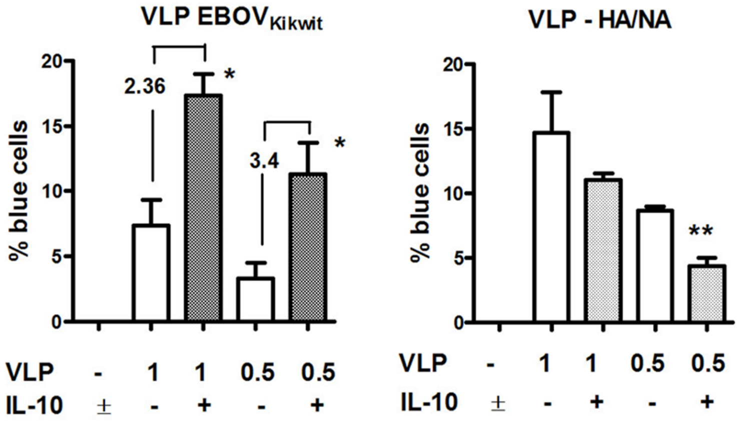

Figure S1. IL-10 has a more pronounced enhancing effect (fold increase) when monocyte-derived macrophages (MDM) are infected with a reduced amount of $EBOV_{Kikwit}$ virus-like particles (VLP). MDM were pre-incubated with IL-10 (20 ng/mL) for 48 h prior to infection with 50µl or 25 µl of $EBOV_{Kikwit}$ VLP or HA/NA VLP, respectively. The cells were processed and the VLP entry/fusion was analyzed by Laser Scanning Cytometry as described in Materials and Methods. No significant difference was observed in the background fluorescence of uninfected IL-10 or mock treated cells. The fold-changes in $EBOV_{Kikwit}$ VLP entry induced by IL-10 when the infection was carried out with different amounts of VLP are indicated in the graph. * $p \leq 0.05$; ** $p \leq 0.01$; ns = not significant.

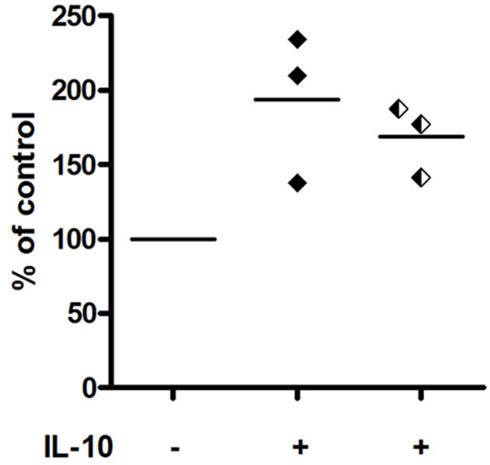

Figure S2. The IL-10 enhancing effect is independent of the type of packaging plasmid used to generate the $EBOV_{Kikwit}$ GP pseudotyped VLPs. VLPs were generated in 293T cells by using either EBOV VP40 or HIV-1 gag-pol (psPAX2)-derived packaging plasmids. BlaM was introduced into the VLPs by co-transfection with the VP40-BlaM or Vpr-BlaM encoding plasmids, respectively. After infection for 3.5 h with $EBOV_{Kikwit}$ GP/VP40/VP40-BlaM (solid diamonds) or $EBOV_{Kikwit}$ GP/psPAX2/Vpr-BlaM VLPs (black and white diamonds), the MDM were washed, loaded with the fluorescent dye CCF2/AM and prepared for analysis by Laser Scanning Cytometry as described in Materials and Methods. The data from the individual experiments, used to generate Figure S2, are provided in Table S1A and Table S1J.

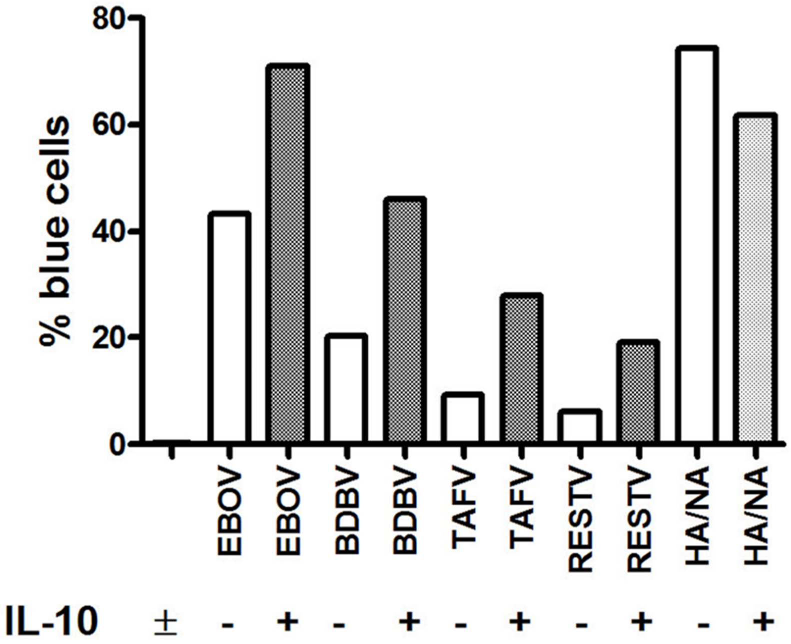

Figure S3. IL-10 enhances fusion of primary MDM with VLPs pseudotyped with envelope glycoproteins from different filovirus species. MDM were pre-incubated with 20 ng/mL IL-10 or DPBS supplemented with 0.5% human serum albumin (HSA) (mock treated) for 48 h in 48-well Nunc tissue culture plates. Subsequently, the cells were infected, loaded and incubated with CCF2/AM fluorescent dye, then detached, fixed with paraformaldehyde and analyzed by Flow cytometry as described in Materials and Methods. The infection with each VLP type was performed in triplicate wells and the cells were combined in one tube after being detached prior to fixing and Flow cytometry analysis. The first column from the left represents mock-infected cells.

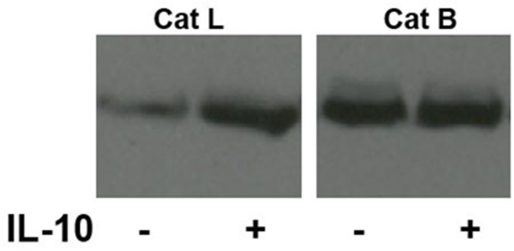

Figure S4. Effect of IL-10 on Cathepsin L expression. MDM were pre-incubated with 20 ng/mL IL-10 or DPBS supplemented with 0.5% HSA (control) for 48 h in 6-well Nunc tissue culture plates. Subsequently, the cells were detached and lysed, and then the cell lysates were analyzed for cathepsin L expression by western blotting as described in Materials and Methods.

Table S2. Significant changes in IL-10-induced gene expression of selected molecules associated with M2c macrophage polarization or EBOV cellular entry.

	Gene	Fold Change 2^(log2FC)
MDM polarization (M2c)	SOCS3	2.716
		2.078
	CD163	3.197
		2.519
	IL21R	2.7
EBOV	Integrin alpha V (ITGAV)	2.133

Cathepsin L (CTSL 1)	2.484<br>1.943
AXL tyrosine kinase (AXL)	1.001
Protein S (PROS 1)	2.732
DC-SIGN (CD209)	-1.130

The mRNA samples from control (mock treated) or IL-10 pre-incubated MDM were prepared and analyzed as described in Materials and Methods. The microarray Illumina Gene Exp. Beadchip used more than one probe for SOCS3, CD163 and Cathepsin L. The molecules listed in Table S1 were selected based on information published in the references cited herein (in the current article).

Table S3. Changes in IL-10 levels observed in survivors and non-survivors during filovirus infection.

IL-10 levels	Species/isolate	Sample/Method	Notes	References
Up to ~7–8 ng/mL (Figure 3)	SUDV/Gulu (2000 outbreak, Uganda)	Serum/Custom Multiplex assay	IL-10 levels higher in fatal infections, compared to survivors up to 7 days after the onset of symptoms, but not in the subsequent interval 8–11 days period)	Hutchinson and Rollin 2007; J Infect Dis 196: S357
Highest mean concentration of ~0.120 ng/mL in patients younger than 21 years of age (Figure 1A)	SUDV/Gulu (2000–2001 outbreak, Uganda)	Serum/Multiplex assay	The highest mean IL-10 concentration was observed during the 0–5 day interval after the onset of symptoms (patients ≤21 years). In fatal cases IL-10 levels remained higher compared to survivors for the monitored period up to 15 days post symptom onset.	McElroy et al. 2014; Emerging Infect Dis 20: 1683 McElroy, Erickson et al. 2014; J Infect Dis 210: 558
0.597 ng/mL median – non-survivors (up to ~7 ng/mL in one of the patients with fatal infection)<br>0.169 ng/mL median – survivors	BDBV (2007 outbreak, Uganda)	Serum or plasma/Multiplex assay	IL-10 levels 3 fold higher in non-survivors compared to survivors. Median time of sample collection for the illness onset 7 days for non-survivors and 7.5 days for survivors<br>In survivors IL-10 was about 20 times higher during 0–11 days after onset of symptoms, compared to convalescent period (35–64 days post onset of symptoms)	Gupta et al. 2012; Virology 432: 119
0.195 ± 0.206 ng/mL fatalities (mean ± SD)<br>0.045 ± 0.033 ng/mL survivors (Table 1)	EBOV/Kikwit (Zaire 1995)	Serum/Enzyme immunosorbent Assays (EIA)	High IL-10 levels associated with fatal infection	Villinger et al. 1999; J Infect Dis 179: S188
0.05 ± 0.01 ng/mL<br>1.02 ± 0.54 ng/mL<br>0.9 ± 0.54 ng/mL<br>1,18 ± 0.32 ng/mL<br>(non-survivors 1, 4, 6 and 8 days post onset of symptoms, respectively)	EBOV/outbreak in Gabon, Feb 1996 and outbreak in Booué-96	Plasma/ELISA	High IL-10 levels associated with fatal infection. Positive correlation with virus antigen titers.<br>In survivors IL-10 levels were 0.07 ± 0.06 ng/mL during 1–4 day period after onset of symptom. Subsequently not different from uninfected controls (<0.02 ng/mL)	Baize, Leroy et al. 2002; Clin Exp Immunology 128: 163
Up to ~2.5–3 ng/mL in some patients (Figure 3)	EBOV/Makona	Plasma/Multiplex assay	Statistically significant association between higher IL-10 levels and disease severity (patients admitted to Emory University Hospital, Atlanta, GA or University of Nebraska Med Center, Omaha, NE)	McElroy et al. 2016; Clin Infect Dis 63: 460
Average levels approximately 0.3 ng/mL in fatalities (up to ~0.9 ng/mL in	EBOV/Makona (Guinea, treatment center	Plasma/Magnetic beads-based Multiplex assay	Statistically significant higher levels of IL-10 in fatal cases compared to survivors.	Ruibal et al. 2016; Nature 533: 460

one of the patients), 0.07–0.08 ng/mL in survivors (Figure 1d)	in Gueckedou and Coyan)			
Average ~ 0.025 ng/mL in non-survivors (up to 0.1 ng/mL in one patent) after 7 days post the onset of symptoms. 0.008–0.009 ng/mL in survivors (Figure S4)	EBOV/Makona (Sierra Leone, late stage of 2014–2015 outbreak (Jan-March 2015)	Serum/ELISA	Higher IL-10 levels correlated with higher virus loads. Significantly higher IL-10 levels in fatal cases compared to survivors after 7 days post the onset of symptoms	Jiang et al. 2017; J Infect Dis 215: 1107
0.25 ng/mL on day 7 of clinical illness. IL-10 levels gradually decreased starting day 8 and returned to normal on day 14 (Fig.S8).	EBOV/Makona	Serum/magnetic beads–based Multiplex assay	Samples from a 34 year old survivor infected in Sierra Leone and evacuated to the National Institutes of Health for treatment on day 7 of clinical illness. Released from the hospital on day 33.	Kash et al. 2017; Sci Transl Med 9: 385
Non-survivors – median IL-10 levels of 0.556 ng/mL (over 3 ng/mL in 5 patients) vs. 0.21 ng/mL for survivors.	EBOV/Makona Guéckédou and Coyah	Plasma/Multiplex assay	In non-survivors the average IL-10 levels started to increase before day 5 post admission, while in survivors, the average IL-10 levels started to decrease before day 5 post admission. The difference in the average IL-10 levels between survivors and non-survivors gradually increased during the duration of the study	Kerber et al. 2018; J Infect Dis 218: S496
